# Screening for cardiac sarcoidosis: diagnostic approach and long-term follow-up in a tertiary centre

**DOI:** 10.1007/s12471-024-01925-0

**Published:** 2025-01-09

**Authors:** Nikki van der Velde, Anne Poleij, Mattie J. Lenzen, Ricardo P. J. Budde, Tessa Brabander, Jelle R. Miedema, Arend F. L. Schinkel, Michelle Michels, Alexander Hirsch

**Affiliations:** 1 Department of Cardiology, Thorax Centre, Cardiovascular Institute, Erasmus Medical Centre, Rotterdam, The Netherlands; 2 Department of Radiology and Nuclear Medicine, Erasmus Medical Centre, Rotterdam, The Netherlands; 3 Department of Respiratory Medicine, Erasmus Medical Centre, Rotterdam, The Netherlands

**Keywords:** Cardiac sarcoidosis, Cardiovascular magnetic resonance imaging, Positron emission tomography, Diagnostic approach

## Abstract

**Background:**

Cardiac sarcoidosis (CS) is associated with poor prognosis, making early diagnosis and treatment important. This study evaluated the results of a diagnostic approach in patients with known sarcoidosis and suspected cardiac involvement in a tertiary centre and their long-term outcomes.

**Methods:**

We included 180 patients with sarcoidosis and a clinical suspicion of CS. In addition to an electrocardiogram (ECG)/transthoracic echocardiogram (TTE), cardiovascular magnetic resonance imaging (CMR) and positron emission tomography (PET) were performed in 66% and 37% of the patients, respectively. The diagnosis of CS was based on the Heart Rhythm Society criteria. Follow-up was performed, and a composite endpoint of sustained ventricular tachycardia, ventricular fibrillation, aborted sudden cardiac death, heart failure hospitalisation, heart transplantation or cardiac death was used for the survival analysis.

**Results:**

Symptoms were present in 87% of the patients, and ECG/TTE abnormalities were found in 92/180 patients (51%). Using CMR and/or PET, 31/92 patients (34%) were diagnosed with CS. In 15 patients, an alternative diagnosis was found. CS was diagnosed in 11/88 patients (13%) without ECG/TTE abnormalities. During a median follow-up time of 4.4 years (interquartile range: 2.3–6.8), 11 composite endpoints occurred, more frequently in CS patients than in sarcoidosis patients without cardiac involvement (*p* < 0.001). Patients with ECG/TTE abnormalities at baseline had worse outcomes than those without abnormalities (*p* = 0.019).

**Conclusion:**

CS was diagnosed in 23% of the referred sarcoidosis patients. ECG/TTE were of limited diagnostic value for screening for CS but seemed to have important prognostic value as patients with normal ECG/TTE results who did meet the diagnostic CS criteria had a very good prognosis. CMR/PET provided a good diagnostic yield and identified other cardiac diseases.

**Supplementary Information:**

The online version of this article (10.1007/s12471-024-01925-0) contains supplementary material, which is available to authorized users.

## What’s new?

- The presence of cardiac symptoms was insufficient to differentiate between sarcoidosis patients with and those without cardiac involvement.
- Patients with a normal electrocardiogram and transthoracic echocardiogram were not exempt from cardiac sarcoidosis (CS), although their prognosis during follow-up was good.
- Cardiovascular magnetic resonance imaging had a good diagnostic yield in the evaluation of suspected CS.
- The prevalence of CS was similar as that reported in other cohort and autopsy studies, even though advanced non-invasive imaging was not standardly performed in each patient.

## Introduction

Sarcoidosis is a systemic inflammatory disease of unknown origin that is characterised by the presence of non-caseating granulomas in various organs including the heart [1, 2]. Symptomatic cardiac involvement occurs in 5% of patients with sarcoidosis, although autopsy studies have shown a prevalence of up to 25% [3–8]. The clinical presentation of cardiac sarcoidosis (CS) varies from an asymptomatic course to overt heart failure (HF) and sudden cardiac death (SCD), and is associated with poor prognosis [9–11]. Therefore, early diagnosis and treatment are important [12–16].

The diagnosis of CS is established by histological diagnosis through an invasive endomyocardial biopsy (EMB) with inherent risks and limited diagnostic yield due to sampling error because of the patchy involvement of the disease [17, 18]. Current guidelines aim to diagnose CS without the necessity of a positive EMB and are based on clinical and diagnostic criteria recommended by experts and limited scientific data [10, 11]. As a result, advanced non-invasive imaging modalities such as cardiovascular magnetic resonance imaging (CMR) and ^18^F‑fluorodeoxyglucose (^18^F‑FDG) positron emission tomography (PET) have been incorporated into these diagnostic guidelines [10, 19, 20]. Although previous research with advanced non-invasive imaging modalities reported similar prevalences of CS as autopsy studies and the sensitivity and specificity of each of these modalities for accurately diagnosing CS have been determined, the most optimal diagnostic approach is still not fully defined [17, 18, 21]. Furthermore, long-term follow-up data of CS patients are limited.

Therefore, the first aim of this study was to evaluate the frequency of CS, and the strengths and limitations of a daily used diagnostic approach for CS in a tertiary centre [10]. Secondly, the long-term outcomes of CS patients based on this diagnostic approach were assessed.

## Methods

### Study population

For this single-centre, observational, retrospective study, we included sarcoidosis patients with confirmed extra-cardiac granulomatous inflammation on extra-cardiac biopsy in accordance with the American Thoracic Society/European Respiratory Society/World Association of Sarcoidosis and other Granulomatous Disorders statement [22] and those with a clinical suspicion of CS who were referred to the outpatient clinic of the cardiology department of the Erasmus Medical Centre in Rotterdam, the Netherlands, from January 2008 through December 2018. Patients were eligible for inclusion if at least an electrocardiogram (ECG) and transthoracic echocardiogram (TTE) were available. According to the Institutional Review Board of the Erasmus Medical Centre, this study did not meet the requirements of a study that is subject to the Medical Research Involving Human Subjects Acts (MEC 2018-1055).

### Clinical assessment

Data on medical history, symptoms, physical examination, ECG and TTE were obtained from the patients’ electronic chart. In case of clinical suspicion of CS, additional tests were performed at the discretion of the treating physician. Diagnostic tests that had already been conducted elsewhere were not routinely repeated. CMR scans that consisted of at least balanced steady-state free precession cine imaging and late gadolinium enhancement (LGE) imaging, and ^18^F‑FDG PET scans that were performed to determine cardiac involvement were assessed or reassessed by our expert radiologists/cardiologists (see Table S1 in Electronic Supplementary Material) and included for analysis [23–25]. Afterwards, each case was screened by 2 authors (NvdV and AH) for the diagnosis of CS based on the Heart Rhythm Society (HRS) criteria [10].

### Outcome measures

Follow-up data were collected until November 2020, which included information on death, hospitalisation for HF, aborted SCD, pacemaker or implantable cardioverter-defibrillator (ICD) implantation, ventricular arrhythmia and heart transplantation. HF was defined as: (1) presence of clinical signs of congestion (including dyspnoea, fatigue, and oedema) requiring diuretics in an outpatient setting or (2) episode of decompensation requiring hospital admission. Aborted SCD was defined as resuscitation after cardiac arrest or appropriate ICD shock. Ventricular arrhythmia was defined as: (1) sustained ventricular tachycardia (VT) lasting ≥ 30 s, (2) ventricular fibrillation or (3) non-sustained VT ≥ 3 beats with frequency ≥ 120 beats per min < 30 s. Clinical data were retrieved from our hospital patient records, and mortality data were retrieved from the civil service population registry.

### Statistical analysis

All continuous data were tested for normality before analysis using the Kolmogorov-Smirnov test and are expressed as mean ± standard deviation or median (interquartile range; IQR), as appropriate. Categorical variables are presented as number (%).

Continuous variables were compared using the Student’s *t*-test or Mann-Whitney U test, and categorical data were compared using the Pearson’s chi-squared test. Kaplan-Meier survival analysis was performed to estimate the cumulative survival for the composite endpoint of sustained VT, ventricular fibrillation, aborted SCD, HF hospitalisation, heart transplantation or cardiac death. To identify independent predictors of the prognosis of these patients, variables were tested in Cox proportional hazard models. First, univariate analysis was performed. Next, a multivariate model (Model 1) was developed using forward stepwise selection (entry *p* = 0.05) using all baseline characteristics except for CMR and ^18^F‑FDG PET parameters. Finally, Model 1 was further adjusted by adding CMR parameters (Model 2) to verify the predictive value of CMR in relation to outcome. Because CMR was only performed in a subset of patients, no model was created that included all parameters. Due to the low number of patients with ^18^F‑FDG PET and events, no multivariate model could be created with these parameters.

All analyses were two-tailed, and *p* < 0.05 was regarded as statistically significant. Statistical analyses were performed using SPSS (version 25; IBM SPSS Statistics, IBM Corporation, Armonk, NY, USA).

## Results

**Fig. 1:**
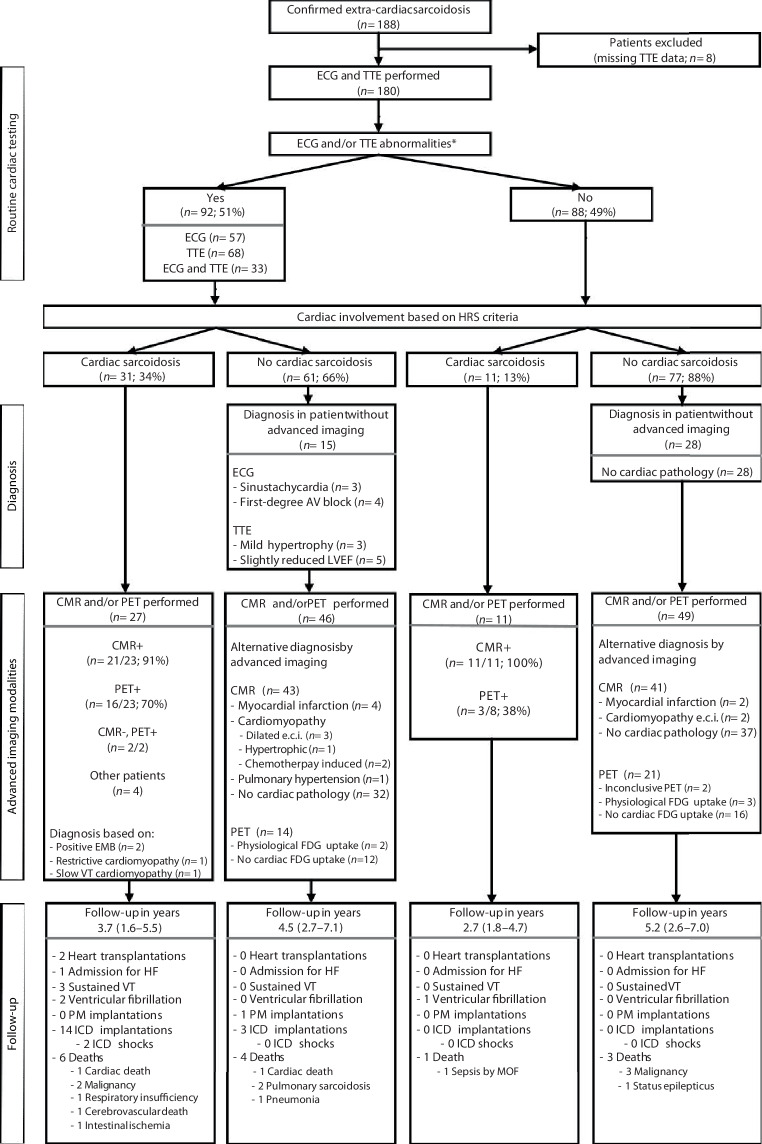
Patient selection, diagnostic approach and median follow-up (interquartile range). *TTE* transthoracic echocardiogram, *ECG* electrocardiogram, *HRS* Heart Rhythm Society, *AV block* atrioventricular block, *LVEF* left ventricular ejection fraction, *CMR* cardiovascular magnetic resonance imaging, *PET* positron emission tomography, *EMB* endomyocardial biopsy, *VT* ventricular tachycardia, *FDG* fluorodeoxyglucose, *HF* heart failure, *PM* pacemaker, *ICD* implantable cardioverter-defibrillator, *MOF* multi-organ failure

A total of 188 consecutive patients with sarcoidosis and clinical suspicion of CS were eligible for this study. Baseline cardiac analysis including ECG and TTE was performed in 180 patients (Fig. 1). Based on the HRS criteria, CS was diagnosed in 42 patients (23%). Baseline patient characteristics stratified by the presence of CS are summarised in Tab. 1. Mean age was 51 ± 11 years, 48% of the patients were male, and most were diagnosed with sarcoidosis stage I or II.

### Diagnostic approach

Figure 1 illustrates how CS was diagnosed. The first step of our diagnostic approach was the assessment of ECG and TTE. Symptoms were not included in the diagnostic algorithm as they frequently occurred in both patients with and without CS (79% vs 89%; *p* = 0.08; Tab. 1). ECG and/or TTE abnormalities were found in 92/180 patients (51%). Details on these abnormalities are shown in Tab. 2. Despite the low absolute numbers of abnormalities, significant differences were found between both groups. ECG and Holter registration showed that CS patients were more likely to have high-degree atrioventricular block, as well as premature ventricular complexes compared with sarcoidosis patients without cardiac involvement. In addition, reduced left and right ventricular ejection fractions and regional wall motion abnormalities on TTE were more common in CS patients. Taken together, ECG and/or TTE abnormalities seemed to be non-specific for CS as they were also present in almost half of the patients without cardiac involvement (61/138; 44%).

**Table 1:** Baseline characteristics of patients stratified by diagnosis of cardiac sarcoidosis based on Heart Rhythm Society criteria

Variable			No cardiac sarcoidosis (*n* = 138)	Cardiac sarcoidosis (*n* = 42)	*P*-value
Age, years			50 ± 12	52 ± 9	0.38
Male gender			65 (47)	21 (50)	0.74
BMI, kg/m^2a^			28 (25–32)	25 (22–29)	0.02
*History*					
	Heart failure		6 (4)	9 (21)	0.002
	Myocardial infarction		4 (3)	0 (0)	0.57
*Therapy*					
	Steroids		55 (40)	23 (55)	0.09
	Other		59 (43)	18 (43)	0.99
*Reason for referral (one or more)*					
	Cardiac symptoms		109 (79)	26 (62)	0.03
	Cardiomyopathy		2 (1)	9 (21)	< 0.001
	ECG abnormalities		21 (15)	12 (29)	0.05
	LGE-positive		2 (1)	1 (2)	0.78
	PET-positive		3 (2)	4 (10)	0.05
	Other reason		28 (20)	17 (40)	0.01
*Symptoms at referral (one or more)*					
	Any symptoms		123 (89)	33 (79)	0.08
		Palpitations	56 (46)	15 (46)	0.99
		Angina pectoris	54 (44)	9 (27)	0.08
		Dyspnoea	71 (58)	19 (58)	0.99
		Near syncope	27 (22)	6 (18)	0.64
		Syncope	10 (8)	2 (6)	1.00
		Other	36 (29)	15 (46)	0.08
*NYHA classification*					
	I		60 (44)	18 (43)	0.94
	II		58 (42)	14 (33)	0.31
	III		19 (14)	7 (17)	0.64
	IV		1 (1)	3 (7)	0.04
*Sarcoidosis*					
	Disease duration before presentation at cardiology outpatient clinic, years		3 (1–8)	1 (0–6)	0.02
	Sarcoidosis Scadding stage				
		0	14 (10)	2 (5)	0.37
		I	51 (37)	14 (33)	0.67
		II	57 (41)	21 (50)	0.32
		III	11 (8)	2 (5)	0.74
		IV	5 (4)	3 (7)	0.39
*Organ involvement*					
	Pulmonary		104 (75)	35 (83)	0.28
	Eyes		27 (20)	6 (14)	0.44
	Skin		31 (23)	6 (14)	0.25
	Lymph nodes		107 (78)	37 (88)	0.13
	Other		41 (30)	17 (41)	0.19
*Diagnostic tests (other than ECG and TTE)*					
	Holter registration		88 (64)	31 (74)	0.23
	CMR		84 (61)	34 (81)	0.02
	^18^F‑FDG PET		35 (25)	31 (74)	< 0.001
	Endomyocardial biopsy		2 (1)	9 (21)	< 0.001

^*18*^*F‑FDG* ^18^F‑fluorodeoxyglucose, *ECG* electrocardiogram, *LGE* late gadolinium enhancement, *PET* positron emission tomography, *NYHA* New York Heart Association, *TTE* transthoracic echocardiogram, *CMR* cardiovascular magnetic resonance imaging

Data are mean ± standard deviation, *n* (%) or median (interquartile range)

^a^Body mass index (*BMI*) was measured in 148/180 patients

**Table 2:** Results of diagnostic tests in patients stratified by diagnosis of cardiac sarcoidosis based on Heart Rhythm Society criteria

Diagnostic test result			No cardiac sarcoidosis (*n* = 138)	Cardiac sarcoidosis (*n* = 42)	*P*-value
*Electrocardiogram*			*(n* = 138)	(*n* = 42)	
AV block present					
		1st or 2nd degree Mobitz type I	11 (8)	3 (7)	0.03
		2nd degree Mobitz type II or 3rd degree	0 (0)	3 (7)	0.03
	Left bundle branch block		4 (3)	3 (7)	0.36
	Right bundle branch block		9 (7)	8 (19)	0.03
	Premature ventricular complex		8 (6)	10 (24)	0.002
	Atrial fibrillation		2 (1)	3 (7)	0.08
	Sinus tachycardia		16 (12)	1 (2)	0.13
*Holter registration*			(*n* = 88)	(*n* = 31)	
	AV block present				
		1st or 2nd degree Mobitz type I	2 (2)	0 (0)	1.00
		2nd degree Mobitz type II or 3rd degree	0 (0)	1 (2)	1.00
	Percentage premature ventricular complex^a^		1 (0–1)	1 (0–2)	0.02
	Non-sustained ventricular tachycardia		10 (11)	8 (26)	0.08
*Transthoracic echocardiogram*			(*n* = 138)	(*n* = 42)	
	LV ejection fraction				
		Normal (> 55%)	110 (80)	19 (45)	< 0.001
		Slightly less (45–55%)	18 (13)	8 (19)	0.33
		Moderate (35–45%)	7 (5)	6 (14)	0.08
		Severely depressed (< 35%)	3 (2)	9 (21)	< 0.001
	LV regional wall motion abnormalities		16 (12)	11 (26)	0.02
	LV hypertrophy		10 (7)	2 (5)	0.74
	TAPSE < 16 mm		4 (3)	7 (17)	0.004
	Valvular dysfunction present		108 (78)	35 (83)	0.48
		≥ Moderate aortic valve insufficiency	3 (3)	0 (0)	1.00
		≥ Moderate aortic valve stenosis	1 (1)	0 (0)	1.00
		≥ Moderate mitral valve insufficiency	3 (3)	5 (14)	0.02
		≥ Moderate pulmonary valve insufficiency	1 (1)	0 (0)	1.00
		≥ Moderate tricuspid valve insufficiency	3 (3)	2 (6)	0.60
*Cardiovascular magnetic resonance*			(*n* = 84)	(*n* = 34)	
	Normal LV function (LV ejection fraction > 50%)		65 (77)	16 (47)	0.001
	Volumetry^b^				
		LV end-diastolic volume, ml	152 (128–195)	175 (144–208)	0.07
		LV end-systolic volume, ml	71 (56–89)	87 (67–134)	0.01
		LV stroke volume, ml	79 (66–97)	78 (62–85)	0.23
		LV ejection fraction, %	54 (51–59)	49 (30–56)	0.004
		LV mass, g	91 ± 29	95 ± 25	0.49
		RV end-diastolic volume, ml	146 (117–191)	160 (128–218)	0.18
		RV end-systolic volume, ml	71 (54–96)	86 (56–125)	0.07
		RV stroke volume, ml	77 (63–98)	76 (61–86)	0.28
		RV ejection fraction, %	53 (47–59)	47 (37–59)	0.02
	Regional wall motion abnormalities^c^				
		LV regional wall motion abnormalities	16 (19)	12 (36)	0.05
		RV regional wall motion abnormalities	2 (2)	5 (15)	0.02
	Late gadolinium enhancement				
		Pattern strongly consistent with CS	0 (0)	21 (62)	< 0.001
		Pattern likely consistent with CS	0 (0)	7 (21)	< 0.001
		Pattern not likely consistent with CS	12 (14)	0 (0)	0.018
		No late gadolinium enhancement	72 (86)	6 (18)	< 0.001
^*18*^*F‑FDG positron emission tomography*			(*n* = 35)	(*n* = 31)	
		Uptake strongly consistent with CS	0 (0)	12 (39)	< 0.001
		Uptake possibly consistent with CS	0 (0)	7 (23)	0.003
		Physiological uptake	5 (14)	1 (3)	0.20
		Inconclusive	2 (6)	2 (6)	1.00
		No uptake	28 (80)	9 (29)	< 0.001
*Endomyocardial biopsy*			(*n* = 2)	(*n* = 9)	
	Positive for sarcoidosis		0 (0)	2 (22)	1.00

*AV block* atrioventricular block, *TAPSE* tricuspid annular plane systolic excursion, *CS* cardiac sarcoidosis, ^*18*^*F‑FDG* ^18^F‑fluorodeoxyglucose

Data are *n* (%), median (interquartile range) or mean ± standard deviation

^a^Percentage of premature ventricular complexes was measured in 50/84 patients and 24/34 patients, respectively

^b^Left ventricular (*LV*) volumes were measured in 83/84 patients and 33/34 patients, respectively, LV mass was measured in 83/84 patients and 32/34 patients, respectively, and right ventricular (*RV*) volumes were measured in 83/84 patients and 32/34 patients, respectively

^c^Wall abnormalities were measured in 83/84 patients and 33/34 patients, respectively

The opposite was also true: a normal ECG and TTE did not exclude patients from having CS as 11 of the 88 patients (13%) without ECG/TTE abnormalities were eventually diagnosed with CS. Most of these patients were referred for further analysis to the cardiologist based on clinical suspicion of cardiac involvement (i.e. symptoms such as angina pectoris, dyspnoea and dizziness) or cardiac arrhythmias (i.e. frequent premature ventricular complexes on Holter registration or during cardiac stress test or atrial and ventricular arrhythmias during hospitalisation).

**Fig. 2:**
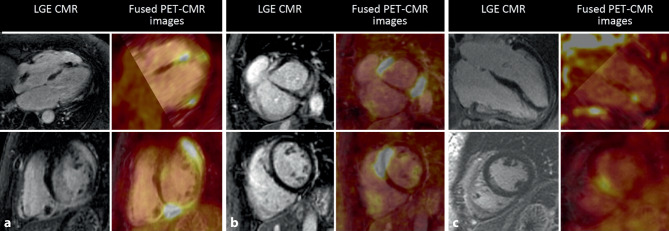
Examples of late gadolinium enhancement (LGE) images and ^18^F‑fluorodeoxyglucose (^18^F‑FDG) positron emission tomography (PET) images with myocardial uptake in 3 patients diagnosed with cardiac sarcoidosis. **a** Extensive mid-myocardial LGE and ^18^F‑FDG uptake inferior and epicardial to mid-wall LGE apicoseptal in 4‑chamber view. Transmural LGE basal anterolateral and inferior with involvement of right ventricle in short-axis view. **b** Mid-myocardial LGE basal inferolateral corresponding to location of ^18^F‑FDG uptake on PET scan. **c** Focal to subendocardial LGE midventricular septum corresponding to location of ^18^F‑FDG uptake on PET scan in short-axis and 4‑chamber views. *CMR* cardiovascular magnetic resonance imaging

Additional testing using CMR and/or ^18^F‑FDG PET was performed in 133/180 patients (74%). The test results are shown in Tab. 2 and Fig. 1. CMR confirmed that reduced systolic function and regional wall abnormalities occurred more frequently in CS patients. LGE and ^18^F‑FDG uptake consistent with CS were also found in these patients (Fig. 2). Advanced non-invasive imaging examinations were also performed in 95/138 patients (69%) without CS, whereby CMR led to an alternative diagnosis (i.e. myocardial infarction or other cardiomyopathies) in 18% of the patients. In 4 CS patients, no additional imaging was performed, and the diagnosis was based on positive EMBs, slow VT in patients with dilated cardiomyopathy and restrictive cardiomyopathy for which other causes had been excluded.

### Long-term outcomes

**Fig. 3:**
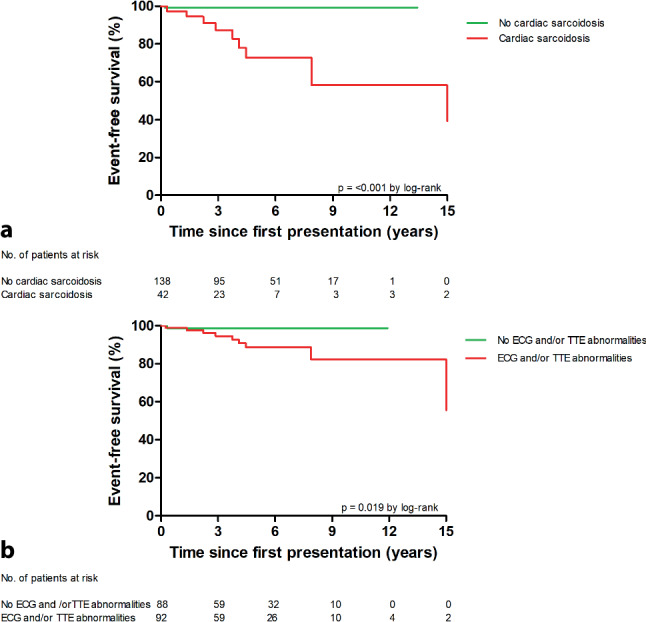
Kaplan-Meier survival analyses showing composite endpoint of sustained ventricular tachycardia, ventricular fibrillation, aborted sudden cardiac death, heart failure hospitalization, heart transplantation or cardiac death. **a** Cardiac-related events in patients with and without cardiac sarcoidosis, **b** Cardiac-related events in patients with and without electrocardiogram (*ECG*) and/or transthoracic echocardiogram (*TTE*) abnormalities

Long-term outcomes are depicted in Fig. 1. A total of 11 composite endpoint events occurred during a median follow-up time of 4.4 years (IQR: 2.3–6.8). CS patients were more likely to experience cardiac-related events than patients without cardiac involvement (log-rank *p* < 0.001; Fig. 3a and see Table S2 in Electronic Supplementary Material). In addition, patients with ECG/TTE abnormalities at baseline had worse outcomes than those without these abnormalities (log-rank *p* = 0.019; Fig. 3b). Especially patients with a normal ECG/TTE had a very good prognosis.

Univariate analysis demonstrated significant associations between the composite endpoint and the presence of left bundle branch block and left ventricular ejection fraction < 45% (Tab. 3). This table also shows the results of various multivariate analyses. In Model 1, which included baseline characteristics, ECG and TTE variables, only left ventricular ejection fraction < 45% on TTE was associated with the composite endpoint. Model 2, for which LGE was added to the multivariate model, showed only LGE consistent with CS on CMR was associated with the composite endpoint.

**Table 3:** Univariate and multivariate analysis for composite endpoint of sustained ventricular tachycardia, ventricular fibrillation, aborted sudden cardiac death, heart failure hospitalization, heart transplantation or cardiac death

Variable	Univariate analysis (*n* = 180)			Multivariate analysis—Model 1 (*n* = 180)^a^			Multivariate analysis—Model 2 (*n* = 118)^b^		
	*HR*	*95% CI*	*P‑value*	*HR*	*95% CI*	*P‑value*	*HR*	*95% CI*	*P‑value*
Age at presentation	1.0	1.0–1.1	0.55	–	–	–			
Male sex	1.0	0.3–3.7	1.00	–	–	–			
NYHA class III or IV	1.3	0.3–6.4	0.74	–	–	–			
Cardiac symptoms	0.5	0.1–2.0	0.36						
Other symptoms	1.2	0.2–6.7	0.82						
Heart failure	5.8	1.4–23.6	0.01						
Presence of any atrioventricular block	0.04	0.0–2597.1	0.58	–	–	–			
Left bundle branch block	7.6	1.7–34.2	0.01	–	–	–			
Right bundle branch block	1.2	0.2–9.7	0.85	–	–	–			
Moderate and severely depressed LV ejection fraction (< 45%) on TTE	16.6	3.4–79.8	< 0.001	9.7	1.8–53.6	0.01	7.3	0.8–69.6	0.09
Regional wall motion abnormalities on TTE	1.7	0.4–8.3	0.50	–	–	–			
Positive LGE consistent with cardiac sarcoidosis	22.8	2.7–195.6	0.004	N/A			10.7	1.1–103.2	0.04
^18^F‑FDG uptake consistent with cardiac sarcoidosis	8.6	1.0–76.7	0.06	N/A			N/A		

^*18*^*F‑FDG* ^18^F‑fluorodeoxyglucose, *HR* hazard ratio, *CI* confidence interval, *NYHA* New York Heart Association, *LV* left ventricular, *TTE* transthoracic echocardiogram, *LGE* late gadolinium enhancement,* CMR* cardiovascular magnetic resonance imaging, *PET* positron emission tomography, *N/A* not applicable

^a^Model 1 (forward conditional) included all patients without CMR or ^18^F‑FDG PET variables

^b^For Model 2, LGE was added to Model 1

## Discussion

The aims of this study were to evaluate the diagnostic approach for CS in a tertiary centre and to assess the long-term outcomes of these patients. The main findings were as follows: (1) cardiac symptoms were found to be insufficient to distinguish between cardiac involvement or not, (2) an ECG and TTE without abnormalities made the diagnosis of CS unlikely but could not completely rule out cardiac involvement, (3) advanced non-invasive imaging contributed to the diagnostic yield of the diagnostic approach, and (4) in CS patients without ECG/TTE abnormalities at baseline had a good prognosis during follow-up.

Given the low sensitivity of an invasive EMB and the lack of a gold standard test to diagnose CS, expert opinion-based guidelines have been developed in which advanced non-invasive imaging modalities play an important role [17, 18, 26]. Although several studies have attempted to determine the optimal diagnostic approach for CS, this is still not fully defined. According to the study by Kouranos et al., the initial step of an approach can be based on the presence or absence of cardiac symptoms and/or ECG abnormalities [18]. One of the main reasons for referring sarcoidosis patients for cardiac analysis in the current cohort was the presence of cardiac symptoms. However, our study showed that the presence of cardiac symptoms was not sufficient to distinguish between patients with and those without cardiac involvement. Despite previous studies showing lower sensitivity and specificity of ECG [18, 21], our approach started with the assessment of ECG/TTE abnormalities. In the majority of our patients (88%) without ECG/TTE abnormalities, cardiac involvement could be excluded using the HRS criteria. However, a normal ECG/TTE could not completely rule out cardiac involvement; advanced non-invasive imaging still established cardiac involvement in 13% of these patients. However, follow-up showed these patients had an overall good prognosis as only 1 of them developed sepsis with multi-organ failure and death as a result.

To assess cardiac involvement of sarcoidosis with greater certainty, biomarkers and advanced non-invasive imaging modalities with higher sensitivity and specificity can be incorporated into the diagnostic approach [10, 18, 21, 26–28]. For example, different studies showed that elevated *N*-terminal pro-brain natriuretic peptide (NT-proBNP) levels were associated with a higher risk of cardiac involvement and troponin levels were correlated with disease activity, with a normalisation of these levels after treatment with corticosteroids. It has also been described that troponin and NT-proBNP levels can predict long-term outcomes. For example, troponin is associated with fatal arrhythmias, and NT-proBNP is a predictor of heart failure [21, 29]. Therefore, these biomarkers may contribute to increased diagnostic accuracy of the further diagnostic approach. Unfortunately, in our retrospective study, biomarker data were not available in a sufficient number of patients. Furthermore, it is known from the literature that CMR is a useful modality in the assessment of CS due to its ability to characterise the myocardium (i.e. oedema, LGE) in a non-invasive manner. As a result, multiple studies have been able to demonstrate similar prevalences of CS by CMR as by autopsy [4, 6, 10, 17, 18, 21]. Our study showed a prevalence rate of 23%, which was achieved in a selected patient population without the standard performance of CMR/^18^F‑FDG PET in each patient. However, clinically silent CS can be missed by applying this approach and may have introduced bias. Therefore, the sensitivity and specificity of both advanced imaging modalities were not calculated. It is likely that if CMR and ^18^F‑FDG PET had been performed in every patient, the incidence of (possible) CS could have been slightly higher.

CMR can also contribute to establishing alternative diagnoses if CS can be excluded. In recent years, research has also shown that CMR can contribute to determining the prognosis of CS and deciding whether and when to start therapy (i.e. immunosuppressive therapy or preventive therapy with an ICD in patients with LGE with a higher risk of sustained VT) [17]. In addition, Vita et al. demonstrated that the diagnostic value of determining CS increased when CMR and ^18^F‑FDG PET results were combined and that this was also of importance for treatment [26]. Although CMR/^18^F‑FDG PET has many possibilities to optimise the diagnostic approach of CS, the availability, costs and expertise needed should be taken into account. Performing advanced cardiac imaging in every patient with extra-cardiac sarcoidosis will be demanding. We still recommend the pulmonary physician screens sarcoidosis patients for cardiac symptoms and refers them to the cardiologist if needed. Based on the clinical presentation and ECG/TTE/biomarkers results, the cardiologist will decide with greater certainty whether further additional testing using advanced cardiac imaging are necessary.

Based on our study, we recommend performing CMR in sarcoidosis patients (with confirmed extra-cardiac granulomatous inflammation on biopsy) who have a high clinical suspicion of CS [29]. CS patients are then monitored by the cardiologist over time, with or without repeated advanced imaging. For patients with normal ECG, TTE, biomarkers and CMR, it is unknown whether and when non-invasive imaging should be repeated. Our recommendation should be applied in case of symptoms, ECG changes or flare-ups of extra-cardiac sarcoidosis. However, we have not investigated this in our current study. No standard routine screening intervals are currently recommended [30]. To validate the findings of this observational single-centre study in a broader, multicentre context, a prospective study should be performed. It is of added value if future studies with a larger population and longer follow-up duration can confirm the findings of a good prognosis in sarcoidosis patients with a normal ECG and TTE. The role of cardiac biomarkers such as NT-proBNP and troponin should be further studied to determine whether they can be used as gatekeeper for additional testing. This may make it easier to decide whether additional imaging is necessary and whether patients can be safely discharged from the cardiology outpatient clinic.

### Study limitations

Some potential limitations of our study can be mentioned. This study is sensitive to selection bias, not only because patients were referred to this tertiary hospital—which is also acknowledged as an national and international sarcoidosis expert centre—but also because patients were only included if there was a clinical suspicion of CS based on symptoms or ECG abnormalities. However, as shown herein, symptoms were not a good parameter to discriminate between cardiac involvement or not. Moreover, advanced cardiac imaging with CMR and/or ^18^F‑FDG PET was not standardly performed in each patient but only at the discretion of the treating physician. It is likely that if CMR and ^18^F‑FDG PET were performed in every patient, the incidence of CS was slightly higher. In addition, our study was a single-centre study with a relatively small sample size, especially a low number of patients with CS.

## Conclusion

Of the patients with sarcoidosis referred for cardiac evaluation to our tertiary centre, 23% were diagnosed with CS. Cardiac symptoms and ECG and/or TTE abnormalities were of limited value for CS screening. However, by adding CMR/^18^F‑FDG PET as advanced non-invasive imaging to our diagnostic approach, a higher diagnostic yield was obtained and other cardiac diseases could be diagnosed in patients without CS. Follow-up showed a good prognosis in CS patients without ECG/TTE abnormalities at baseline.

## Supplementary Information

Table S1 Definitions of abnormalities within baseline cardiac analysis and advanced non-invasive imaging modalities

Table S2 Endpoints stratified according to the presence or absence of cardiac sarcoidosis based on the Heart Rhythm Society criteria
