# Supplementary material for: Screening for cardiac sarcoidosis: diagnostic approach and long-term follow-up in a tertiary centre
Source: Neth Heart J. 2025 Jan 9;33(2):55–64. doi: 10.1007/s12471-024-01925-0 (PMC11757833; doi:10.1007/s12471-024-01925-0)
Supplement: Supplementary file 1 — Table S1 Definitions of abnormalities within baseline cardiac analysis and advanced non-invasive imaging modalities [file 12471_2024_1925_MOESM1_ESM.docx]

**Table S1** Definitions of abnormalities within baseline cardiac analysis and advanced non-invasive imaging modalities

| Cardiac symptoms | Palpitations  Angina  Dyspnea  Syncope or near-syncope |
| --- | --- |
| Electrocardiography | Conduction disorders  Left or right bundle branch block  Any atrioventricular block  Supraventricular arrhythmias  Sinus tachycardia ≥100 beats per minute  Atrial fibrillation |
| Transthoracic echocardiography | Left ventricular ejection fraction <55%  Presence of regional wall motion abnormalities  Left ventricular hypertrophy |
| Cardiovascular magnetic resonance imaging | Left ventricular ejection fraction <50%  Presence of regional wall motion abnormalities  The presence of late gadolinium enhancement consistent with cardiac sarcoidosis |
| Positron Emission Tomography | ^18^F-Fluordeoxyglucose uptake consistent with cardiac sarcoidosis |
