# Supplementary material for: Screening for cardiac sarcoidosis: diagnostic approach and long-term follow-up in a tertiary centre
Source: Neth Heart J. 2025 Jan 9;33(2):55–64. doi: 10.1007/s12471-024-01925-0 (PMC11757833; doi:10.1007/s12471-024-01925-0)
Supplement: Supplementary file 2 — Table S2 Endpoints stratified according to the presence or absence of cardiac sarcoidosis based on the Heart Rhythm Society criteria [file 12471_2024_1925_MOESM2_ESM.docx]

**Table S2** Endpoints stratified according to the presence or absence of cardiac sarcoidosis based on the Heart Rhythm Society criteria

| **Variables** | **No cardiac sarcoidosis**  **(n=138)** | **Cardiac sarcoidosis**  **(n=42)** |
| --- | --- | --- |
| Composite endpoint | 1 | 10 |
| Cardiac death | 1 | 1 |
| Heart transplantation | 0 | 2 |
| Ventricular fibrillation | 0 | 3 |
| Sustained ventricular tachycardia | 0 | 3 |
| Aborted sudden cardiac death | 0 | 0 |
| Heart failure hospitalization | 0 | 1 |
| ICD implantation | 3 | 14 |
| Death | 8 | 7 |

* Composite endpoint of sustained ventricular tachycardia, ventricular fibrillation, aborted sudden cardiac death, heart failure hospitalization, heart transplantation or cardiac death
